# Veratrum Viride in Acute Mania a Potu

**Published:** 1864-08

**Authors:** C. R. Harris

**Affiliations:** Brandy Station, Culpeper County, Virginia.


ART. III.—*Veratrum Viride in Acute Mania a Potu.* By C. R. HARRIS, M. D., Brandy Station, Culpeper county, Virginia.

Having read with great interest and pleasure in Vol. I., Art. 2d, of the "Confederate States Medical and Surgical Journal," February, 1864, an able and valuable communication on the subject of Hydrocyanic Acid in the Treatment of

Insanity, by Dr. Kenneth McLeod, I am induced to report a case of Acute Mania a Potu, which came under my care two or three years ago, and which, I hope, may not prove uninteresting to our profession.

It appears, in the treatment of forty cases of insanity by Dr. McLeod, in which he used the hydrocyanic acid, thirteen were acute mania. The symptom which indicated the administration of the drug as a calmative, was cerebral excitement; in all the other cases reported, occurring in chronic mania, *i. e.*, chronic mania with acute paroxysms, with puerperal epileptic menstrual excitement, etc.

We are forced to admit, from the report of Dr. McLeod, that remarkable success attended the administration of his remedy. The profession are united in relation to the *modus operandi* of hydrocyanic acid. That it is a well known and powerful nervous sedative, all admit.

What was the remedial power excited by the remedy in the patients treated by Dr. McLeod? Reasoning *a priori* it was sedative and calmative, thus allaying cerebral excitement and at the same time exerting its peculiar salutary influence upon the nervous system by curbing and controlling the heart's action.

My patient, to whom I have heretofore alluded, was a case of acute and violent mania a potu, from the excessive use of alcohol for a period of six or eight days. He was generally of temperate habits, and for months a strict "teetotaller." Prior to this attack, he had used little or no alcohol potations of any kind for several weeks.

Becoming worried, as I learned from him, in regard to some unpleasant business transactions with a neighbor, he resorted to free and excessive use of stimulus for the period mentioned, not at any time to full drunkenness, but to an inordinate degree of excitement. He was of full, vigorous habit, stout, athletic and healthy, of a well-marked, sanguineous temperament, and a most powerful muscular development.

He had been laboring under intense mental aberration in violent paroxysms, occurring every hour, or more frequently, during the twenty-four hours preceding the time when I was called to visit him.

The violent paroxysmal attacks were marked by an intensified action of the brain, with a strong disposition to destroy the lives of those he loved most. With the aid of a powerful athletic servant or two, I was enabled to restrain him until there was a decided and happily marked change in his condition. His symptoms were striking:—Eyes suffused, wild, glaring and fiendish expression, with intense excitement (when composed or quiet in bed) of the carotids; pulse full and over 100; skin hot and dry; arrest of all the important secretions. Viewing his case as a critical one and exceedingly unpleasant, I requested a consultation. After the messenger was dispatched, his symptoms were aggravated, and there seemed to be little or no hope for bringing into play any remedial efforts or treatment.

He refused to submit to treatment of any kind. Cold applications to the head were resisted under a threat if we applied them, although he complained of pain in the orbital region. His voice was husky and throat dry. Seeing some chance of deception by the administration of Norwood's veratrum viride, I asked him if he would take a few drops or teaspoonful of honey, to which he readily assented, as he said he knew the honey had no poison in it. Eluding his observation, after an awful paroxysm, in which he was well nigh exhausted, including the two strong servants and myself, I prescribed, pro-renata, eight drops of Norwood's tinct. in the honey, as I said to him, for his dry, hacking cough and hoarseness, both the result of inordinate use of the vocal organs, in swearing, screaming and hallooing. Waiting near an hour and seeing no result whatever, I again administered ten drops as before. He had one paroxyism shortly after, the most awful I ever witnessed. In twenty minutes afterwards he remarked to me, "Doctor, I am awful sick and weak, and am going to die." I at once recognized the peculiar effects of my remedy with great satisfaction—for hours I had been contending with the superhuman efforts of one of the most powerful frames in Virginia. The countenance had changed into calm serenity, a marked pallor was visible on the features; pulse 65, soft and compressible; reason was enthroned, an amiable temper, a kind neighbor, a doating husband and parent was recognized again. In this condition he remained for an hour, conversing rationally, complaining of nausea and some prostration. I ordered a little ginger tea with a small quantity of spirits. He remarked to me that he felt inclined to sleep. I ordered out the servants, and taking my seat by his bed, joyfully watched the approach of Morpheus, which happened in twenty minutes. He slept sweetly and naturally for seven hours. When he awoke, he was well. I ordered him tea and toast, and in a few days he was out and attending to his business. He seemed to recollect nothing of what occurred during the time of his aberration, which was near two days. I gave him nothing more in the shape of medicine, but directed some attention to diet, allowing him food freely, nutritious but digestible.

In this patient we had utter delirium, marked by intense cerebral excitement, with incoherence and delusions of all sorts and degrees. He had active propensities which were destructive, malevolent and homicidal.

What remedy would have so happily, promptly, efficiently and safely relieved the symptoms in this case? It was the only available remedy. It is a well known, and, in the instance alluded to, proved a precious, sedative.

My report has been more prolix than I desired.

The interesting and valuable article of Dr. McLeod, and my opinion relative to the philosophy of his treatment, has strengthened my confidence in the course I pursued, and which was fully sustained by my friend, Dr. R., who arrived after the patient was well, and with whom I wished to have a consultation. I should not hesitate hereafter, in a similar case, as to the course I would pursue. In acute inflammation of the brain or its membranes, especially involving the arachnoid, not, however, to the exclusion of the lancet and other adjuncts, I doubt not the veratrum viride or Dr. McLeod's dilute hydrocyanic acid, by Scheele, would prove a valuable remedy. I do not now allude to any complications of the cerebral mass induced by alcohol, but ordinary sporadic attacks of acute meningitis or cerebritis.

